# Advances in blood–brain barrier modeling in microphysiological systems highlight critical differences in opioid transport due to cortisol exposure

**DOI:** 10.1186/s12987-020-00200-9

**Published:** 2020-06-03

**Authors:** Jacquelyn A. Brown, Shannon L. Faley, Yajuan Shi, Kathleen M. Hillgren, Geri A. Sawada, Thomas K. Baker, John P. Wikswo, Ethan S. Lippmann

**Affiliations:** 1 Vanderbilt Institute for Integrated Biosystems Research and Education, Vanderbilt University, Nashville, TN USA; 2 Department of Biomedical Engineering, Vanderbilt University, Nashville, TN USA; 3 Department of Chemical and Biomolecular Engineering, Vanderbilt University, Nashville, TN USA; 4 Departments of ADME and Toxicology, Eli Lilly and Company, Indianapolis, IN USA; 5 Department of Molecular Physiology and Biophysics, Vanderbilt University, Nashville, TN USA; 6 Department of Physics, Vanderbilt University, Nashville, TN USA; 7 Vanderbilt Brain Institute, Vanderbilt University, Nashville, TN USA

## Abstract

**Background:**

The United States faces a national crisis involving opioid medications, where currently more than 130 people die every day. To combat this epidemic, a better understanding is needed of how opioids penetrate into the central nervous system (CNS) to facilitate pain relief and, potentially, result in addiction and/or misuse. Animal models, however, are a poor predictor of blood–brain barrier (BBB) transport and CNS drug penetration in humans, and many traditional 2D cell culture models of the BBB and neurovascular unit have inadequate barrier function and weak or inappropriate efflux transporter expression. Here, we sought to better understand opioid transport mechanisms using a simplified microfluidic neurovascular unit (NVU) model consisting of human brain microvascular endothelial cells (BMECs) co-cultured with astrocytes.

**Methods:**

Human primary and induced pluripotent stem cell (iPSC)-derived BMECs were incorporated into a microfluidic NVU model with several technical improvements over our previous design. Passive barrier function was assessed by permeability of fluorescent dextrans with varying sizes, and P-glycoprotein function was assessed by rhodamine permeability in the presence or absence of inhibitors; quantification was performed with a fluorescent plate reader. Loperamide, morphine, and oxycodone permeability was assessed in the presence or absence of P-glycoprotein inhibitors and cortisol; quantification was performed with mass spectrometry.

**Results:**

We first report technical and methodological optimizations to our previously described microfluidic model using primary human BMECs, which results in accelerated barrier formation, decreased variability, and reduced passive permeability relative to Transwell models. We then demonstrate proper transport and efflux of loperamide, morphine, and oxycodone in the microfluidic NVU containing BMECs derived from human iPSCs. We further demonstrate that cortisol can alter permeability of loperamide and morphine in a divergent manner.

**Conclusions:**

We reveal a novel role for the stress hormone cortisol in modulating the transport of opioids across the BBB, which could contribute to their abuse or overdose. Our updated BBB model represents a powerful tool available to researchers, clinicians, and drug manufacturers for understanding the mechanisms by which opioids access the CNS.

## Introduction

The blood–brain barrier (BBB) consists of brain microvascular endothelial cells (BMECs) that are surrounded and supported by astrocytes and pericytes. It plays critical roles in brain homeostasis and neural function by regulating the transfer of substances from the peripheral circulation into the brain [1, 2]. The endothelial cells of the brain capillaries form a continuous/non‐fenestrated membrane comprised of specialized tight junctions that limit passive transport [3, 4]. The BBB further controls penetration into the central nervous system (CNS) with P-glycoprotein efflux transport which is highly critical for regulating neuropharmacokinetics and neuropharmacology [5]. In addition, the BBB serves as a metabolic barrier with transport and efflux systems embedded within both luminal and abluminal membrane surfaces, which enables proper waste and nutrient processing [6]. Thus, the BBB serves as a selective gatekeeper to the CNS by limiting paracellular diffusion, suppressing transcytosis, and selectively controlling molecular transport [1, 7–9]. These features enable and contribute to the restricted brain penetration of a number of substances and thus facilitate a highly regulated CNS environment necessary for proper neuronal function.

Opioids must cross the BBB to exert their analgesic effects in the CNS. As opioids are typically small hydrophobic molecules that can readily diffuse into a lipid bilayer, their penetration through the BBB depends primarily on whether the compound is a substrate for an efflux transporter. For example, oxycodone is incredibly potent in part because it is not recognized by any of the major BBB efflux transporters [10] and may be actively imported by nutrient transporters [11, 12]. Morphine is a substrate for P-glycoprotein [13] whereas its primary metabolites, which also have analgesic potency, are not believed to be P-glycoprotein substrates but may bind other transporters [14]. Meanwhile, loperamide, a synthetic opioid receptor agonist that is used clinically as an anti-diarrheal, strongly binds P-glycoprotein and therefore is excluded from the CNS [15, 16], which is important for its marketed use for inducing opioid-mediated constipation in the gastrointestinal tract. Overall, the basic mechanisms of opioid transport across the BBB have been intensely studied [17]. However, very little is known about how endogenous biological cues may influence opioid penetration into the CNS by modulating BBB properties. A better understanding of any potentially biological factors that could influence opioid transport across the BBB is paramount for preventing and treating opioid abuse and overdose.

While CNS opioid penetration can be studied using animals, interspecies differences in BBB transporter homology and expression can limit the predictive power of assays performed in vivo [18, 19]. Moreover, it is difficult to comprehensively study transport mechanisms in animals due to the limited throughput of in vivo experiments. For this reason, in vitro models of the human BBB are frequently employed to increase experimental throughput and facilitate interrogation of a wider range of biological variables in a human-relevant system. Such models can range from simple Transwell setups to more complex microfluidic chips, where BMECs are separated from supporting neurovascular cells by a semi-permeable membrane and transport of compounds across the endothelial monolayer can be quantitatively assessed under more realistic physiological parameters (e.g. fluidic shear) [20]. Cells can be obtained from primary, immortalized, or induced pluripotent stem cell (iPSC) sources, with experimental tradeoffs in each case depending on the use of the model system [21]. Intriguingly, despite recent advancements in human in vitro models of the BBB, very few studies have focused on opioid transport across human BMECs.

In this manuscript, we sought to investigate and compare opioid transport using our previously described microfluidic model. We first benchmarked improvements to our previously described microfluidic system [22, 23] using primary human BMECs; these technical and methodological refinements yielded a microfluidic BBB model with reduced experimental variability and increased passive barrier function relative to traditional Transwell setups. We next demonstrate that transport of three opioids with varying sensitivity to efflux (loperamide, morphine, and oxycodone) is appropriately recapitulated when the model is constructed with human iPSC-derived BMECs [24, 25]. Last, we demonstrate that the stress hormone cortisol can unexpectedly influence opioid transport without additional external manipulation of efflux activity. Collectively, our findings demonstrate the applicability of a microfluidic human BBB model for studying opioid transport and indicate that endogenous blood-born cues can impact opioid penetration through BMECs.

## Materials and methods

### NeuroVascular Unit microfluidic device assembly

**Fig. 1:**
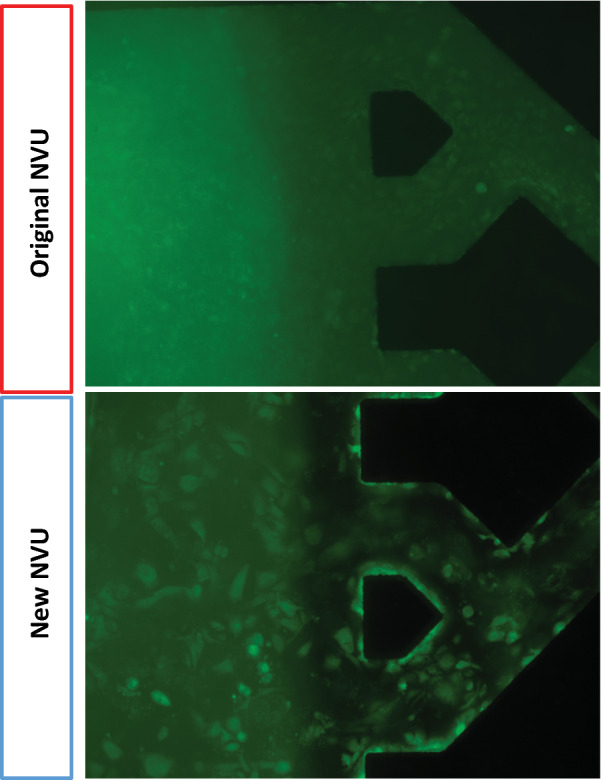
Fluorescent imaging of primary human BMECs in the original versus new NVU design. Images were acquired 3 days after seeding and are representative of multiple devices. The transition from a polycarbonate membrane in the original NVU to a PET membrane (which is thinner and more optically transparent) in the updated NVU improves the ability to image cells in real-time

The NeuroVascular Unit (NVU) microfluidic device was fabricated by the Vanderbilt Institute for Integrative Biosystems Research and Education (VIIBRE) Microfabrication Core as previously described in [23]. In brief, the basic design of the device is a two-chamber system divided by a porous membrane. Relative to our old design, we now use Poly(ethylene glycol) bis(amine) (Sigma-Aldrich, St. Louis, MO, USA) treatment to both bond the porous membrane in place and to modify the surface chemistry for better extracellular matrix (ECM) adsorption, which helps improve cellular attachment. We also transitioned from a 0.4 μm pore diameter polycarbonate membrane to a 3 μm pore diameter polyethylene terephthalate (PET) membrane, which improves phase imaging of the NVU (Fig. 1). Similar to the previous design, each chamber has its own inlet and outlet for perfusion. The device can be perfused in any orientation, so that cells seeded into a chamber can be induced by gravity to adhere to one side of the porous membrane and, by inverting the device and seeding the opposite side with a different cell type, they can be grown in opposition to one another to form the BBB [23]. As previously described, the perfusion system that feeds the brain chamber has a series of switch backs and thus allows a mild media exchange without the introduction of shear stress into the brain chamber [23]. Perfusion was established via syringe pumps where syringes were exchanged every 6 days or 24 h prior to drug permeability assays.

### Cell culture

#### Induced Pluripotent Stem Cell (iPSC) maintenance

CC3 iPSCs were maintained using E8 medium and growth factor-reduced Matrigel (Corning, Corning, NY, USA) coated 6-well plates as described previously [26]. Cells were passaged with Versene (ThermoFisher Scientific, Waltham, MA, USA) when 60–80% confluence was reached and replated at a 1:6 ratio in E8 medium supplemented with 10 µM Y-27632 [27].

#### Differentiation of iPSCs to BMECs

BMECs were differentiated from CC3 iPSCs according to previously reported protocols, with minor modifications [26, 27]. Briefly, CC3 iPSCs were seeded at a concentration of 150,000 cells per well of a Matrigel-coated 6-well plate in E8 with 10 µM Y-27632. The following day, medium was replaced with E6 medium (ThermoFisher Scientific) and changed daily for 4 days. On day 4, the medium was changed to Neurobasal (Gibco) supplemented with 200× B27 (Gibco), 0.5 mM l-glutamine (ThermoFisher Scientific), 20 ng/ml basic fibroblast growth factor (bFGF; Peprotech, Rocky Hill, NJ, USA), and 10 μM all-trans retinoic acid (RA; ThermoFisher Scientific) for 48 h. BMECs were subcultured on day 6 in the same medium also containing 10 µM Y-27632. For traditional experiments, BMECs were subcultured onto PET Transwells of 3 µm pore size or plastic plates that were coated with 400 ng/ml collagen IV (Sigma-Aldrich) and 100 ng/ml fibronectin (Sigma-Aldrich) in 1× PBS (surfaces were coated at 37 °C for 24 h prior to seeding). After 24–48 h, medium was changed to Neurobasal with 200× B27 and 0.5 mM l-glutamine.

#### Primary cell culture

Primary cell culture was carried out as previously described [23]. Primary human BMECs were purchased from Cell Systems (Kirkland, WA, USA) and primary human astrocytes were purchased from ATCC (Manassas, VA, USA). BMECs were maintained in EBM-2 medium (Lonza, Basel, Switzerland). Astrocytes where maintained in Neurobasal supplemented with 50× B27. After receipt from the vendor, primary BMECs and astrocytes were thawed, expanded for three passages, and banked as frozen stocks in complete media supplemented with 10% DMSO. A single vial of cells was then thawed and allowed to recover for 3–4 days before being seeded into either the NVU device or a Transwell system.

#### NVU cell culture

NVUs were seeded with BMECs (either primary or iPSC-derived) in the lower chamber and astrocytes in the upper chamber. Before endothelial cells were introduced into the lower vascular chamber of the NVU, the device was coated with 10 µg/ml poly-d-lysine (Sigma-Aldrich) for 4 h at 37 °C, then coated with 400 ng/ml collagen IV and 100 ng/ml fibronectin for 24 h at 37 °C. On day 0, BMECs were loaded into the vascular chamber at a density of (5–8) × 10^7^ cells/ml, followed by device inversion to allow cell attachment to the membrane. On day 1, the device was returned to its original orientation and astrocytes were loaded into the upper brain chamber at a density of 2 × 10^6^ cells/ml and given 24 h for attachment. On day 2, media was perfused through both chambers at a rate of 2 µl/min. Primary BMECs were perfused with EBM-2 and iPSC-derived BMECs were perfused with Neurobasal supplemented with 200× B27 and 0.5 mM l-glutamine, whereas the brain chamber containing astrocytes was always perfused with Neurobasal supplemented with 200× B27 and 0.5 mM l-glutamine. Devices containing primary BMECs were maintained for 8–15 days to establish barrier function, whereas devices containing iPSC-derived BMECs were maintained for 24–48 h before experiments were conducted.

#### Immunocytochemistry

Cells were prepared for immunofluorescence analysis based upon methods reported previously [24, 25]. Briefly, iPSC-derived BMECs cultured in 12-well plates were washed with 1× PBS and fixed for 10 min in 4% paraformaldehyde (Sigma-Aldrich) or BD Cytofix (Becton-Dickenson) and blocked at 4 °C overnight in PBSG [1× PBS containing 5% goat serum (ThermoFisher Scientific)]. Antibodies utilized in this study were: anti-ZO-1 (ThermoFisher Scientific, clone ZO1-1A12, 1:100 dilution), anti-claudin-5 (ThermoFisher Scientific, clone 4C3C2 Alexa Fluor 488 conjugate, 1:100 dilution), and anti-VE-cadherin (R&D Systems, AF938 goat polyclonal, 1:100 dilution). Cells were incubated with primary antibody diluted in PBSG overnight at 4 °C. Cells were then washed five times with PBSG (minimum wash time of 5 min) and incubated with appropriate secondary antibodies diluted at 1:1000 in PBSG for a minimum of 2 h at room temperature. Following secondary incubation, cells were washed with 1× PBS five times prior to visualization with a Zeiss EVOS imaging system.

### Passive permeability measurements in NVUs

A host of dextrans were used to evaluate passive permeability: a 40 kDa FITC dextran, a 10 kDa Cascade Blue dextran, a 70 kDa Texas Red dextran, and a 3 kDa Alexa Fluor 680 dextran (ThermoFisher Scientific). Stock solutions were made in water at 1 mM and stored at − 20 °C. Working concentrations were prepared at 1 µM in BMEC culture media (tailored to either primary or iPSC-derived BMECs). As previously described [23], the vascular compartment of the NVU was perfused with dextran solution for the entire duration of culture. For passive permeability measurements, effluent was collected from the brain compartment over a fixed amount of time and analyzed for fluorescence intensity using a plate reader (Tecan M1000). By measuring concentration in the brain compartment, we calculated the applied permeability coefficient as

$$P_{app} { = }\frac{{V_{b} \times C_{a} }}{{C_{b} \times A \times t}}$$

where V_b_ is the brain chamber volume in cm^3^, A is the membrane growth area in cm^2^, C_a_ is the initial vascular concentration of dextran in µM, C_b_ is brain concentration of dextran in µM, and t is the assay time in seconds. The effective permeability of the BMEC monolayer was calculated by subtracting the permeability of an empty device according to the equation:

$$\frac{1}{{P_{total} }} = \frac{1}{{P_{cells} }} + \frac{1}{{P_{membrane} }}.$$

In some experiments where comparisons were not made to Transwells, we reported permeability as percent transport, which is the ratio of each compound in the brain chamber relative to the vascular chamber.

### Passive permeability measurements in Transwells

The apical Transwell chamber media was replaced with 250 µl of maintenance media containing 1 µM fluorescent dextran as described above. At 30 min intervals over a 2 h period, 200 μl of media from the basolateral chamber was collected and replaced with fresh maintenance media. Fluorescence of collected media was measured using a plate reader in order to determine concentration of dextran crossing the BMEC monolayer. Permeability was calculated as

$$P_{app} { = }\frac{{V_{b} \times C_{a} }}{{C_{b} \times A \times t}},$$

where V_b_ is the basolateral volume in cm^3^, A is the membrane growth area in cm^2^, C_a_ is the initial apical concentration in µM, C_b_ is basolateral concentration in µM, and t is the assay time in seconds. The effective permeability of the BMEC monolayer was calculated by subtracting the permeability of the empty Transwell membrane according to the equation:

$$\frac{1}{{P_{total} }} = \frac{1}{{P_{cells} }} + \frac{1}{{P_{membrane} }}.$$

### Efflux transporter activity in NVUs and Transwells

To assess efflux transporter function in BMECs cultured on Transwell filters, BMECs were pre-incubated with either 10 μM cyclosporin A (Tocris, Minneapolis, MN, USA) or 10 μg/ml LSN335984 (Eli Lilly and Company, Indianapolis, IN, USA) in the apical chamber for 1 h. Medium in the apical chamber was then replaced with 250 μl of standard culture medium supplemented with 10 µM Rhodamine 123 (ThermoFisher Scientific). The same calculations described above were used to determine Rhodamine 123 permeability across the BMECs with and without P-glycoprotein inhibition.

To assess efflux transporter function in BMECs cultured in NVU devices, either 10 μM cyclosporine A or 10 μg/ml LSN335984 were added to standard culture medium and perfused over the BMECs for 24 h. 10 µM Rhodamine 123 in standard culture medium was then perfused across the BMECs for 1 h. The same calculations described above were used to determine Rhodamine 123 permeability across the BMECs with and without P-glycoprotein inhibition.

### Opioid transport in NVUs

Morphine, oxycodone and loperamide were purchased from Sigma-Aldrich and applied to the vascular chamber at 41 µg/ml (143.7 µM) morphine, 10 µg/ml (31.7 µM) oxycodone, or 1.5 µg/ml (3.1 µM) loperamide. These concentrations were chosen as those closest to physiological that could still be reliably detected after transport [17]. Each drug was perfused through the vascular chamber for 24 h to establish equilibrium. Perfusion with drug was then continued over a 1 h period, with active sample collection from both the vascular and brain chambers (120 µl total from each compartment). Samples were stored at − 80 °C until processing for mass spectrometry analysis. Opioid transport was calculated as the amount of opioid detected in the brain chamber compared to the total amount of opioid delivered to the vascular chamber (percent of opioid transported across the BMEC monolayer).

### Mass spectrometry sample preparation and opioid detection

The samples were prepared by taking a 50 µl aliquot of frozen sample and adding 300 µl of acetonitrile and 10 μl of internal standard (1 μg/ml of morphine-d3 [Cerriliant, Round Rock, TX, USA)]. Samples were then placed in a speedvac and dried before being reconstituted in 50 µl of the starting buffer (95% H_2_O, 5% acetonitrile).

#### Morphine and oxycodone

The chromatographic system consisted of two 1260 binary pumps (Agilent Technologies, Santa Clara, CA, USA), a 1260-degasser, a thermostatted column compartment (TCC), an SL temperature-controlled column compartment, and a temperature-controlled 1260 HiP-ALS auto-sampler, kept at 4 °C. Separation of a 10 µl aliquot of the sample was achieved using an Agilent Poroshell 120 EC-18 (3.0 mm × 50 mm 2.7 micron) column (Agilent Technologies). The temperature of the column compartment was set to 40 °C. The injection needle was repeatedly rinsed with a 1:1 H_2_O:MeOH solution after each injection. A gradient of 5 mM ammonium formate in water (mobile phase A) and 0.1% formic acid in acetonitrile (mobile phase B) was used. The instrument is a 6430 triple quadrupole mass spectrometer (Agilent Technologies) with an API-Electrospray source operated in positive ion mode. The mass spectrometer was operated in multiple-reaction monitoring (MRM) mode. The source conditions were chosen to give satisfactory signal for all analytes and are as follows: gas temperature = 325 °C; gas flow = 10 l/min; nebulizer = 40 psi; capillary voltage = positive 4000 V, negative 3500 V. This instrument was operated with the Mass Hunter data acquisition software, and data was processed using Quantitative Analysis B.04.00/Build 4.0.225.19 (Agilent Technologies). Morphine was monitored with an MRM transition of 286.3 to 165.1 with a fragmentor setting of 150 and collision energy of 41, and oxycodone (316.3 to 241.1) used a fragmentor setting of 120 and collision energy of 29.

#### Loperamide

The chromatographic system consisted of an Acquity HPLC Binary Solvent Manager, a temperature controlled sample manager kept at 4 °C, and a thermostatted column compartment (Waters, Milford, MA, USA). Separation of a 10 µl aliquot of the sample was achieved using an Agilent Poroshell 120 EC-18 (3.0 mm × 50 mm 2.7 micron) column. The temperature of the column compartment was set to 40 °C. The injection needle was repeatedly rinsed with a 1:1 H_2_O:MeOH solution after each injection. A gradient of 0.1% formic acid in 95% H_2_O/5% acetonitrile (mobile phase A) and 0.1% formic acid in 5% acetonitrile/95% H_2_O (mobile phase B) was used. The instrument used is a TSQ Vantage triple quadrupole mass spectrometer (ThermoFisher Scientific) with a heated ESI source operating in SRM mode. The source conditions optimized for loperamide are as follows: Spray voltage = 3000 V; sheath gas = 30; aux gas = 10; capillary temperature = 300 °C. The instrument was controlled through the Xcalibur software and the data was processed using LCQuan (ThermoFisher Scientific). The loperamide SRM transition that was monitored was 477.1 to 266.5 with a collision energy of 15.

### Evaluating impact of cortisol upon opioid transport in NVUs and Transwell filters

Cortisol was purchased from Sigma-Aldrich under the brand name hydrocortisone and reconstituted in ethanol as a 1.1 mM stock solution. Hydrocortisone was then diluted 1:1000 in culture medium and either perfused through the vascular chamber of the NVUs or added to the apical chamber of the Transwell filters for 24 h. Opioid transport experiments were then carried out as described above. This in vitro concentration of cortisol is 36 µg/dl, which is in the range of cortisol levels associated with pain or trauma [28, 29], whereas normal cortisol levels are estimated around 10–23 µg/dl [30].

## Results

### Advances in device fabrication and cell culture accelerate passive barrier formation and increase barrier strength in primary human BMECs cultured in the microfluidic NVU model

**Fig. 2:**
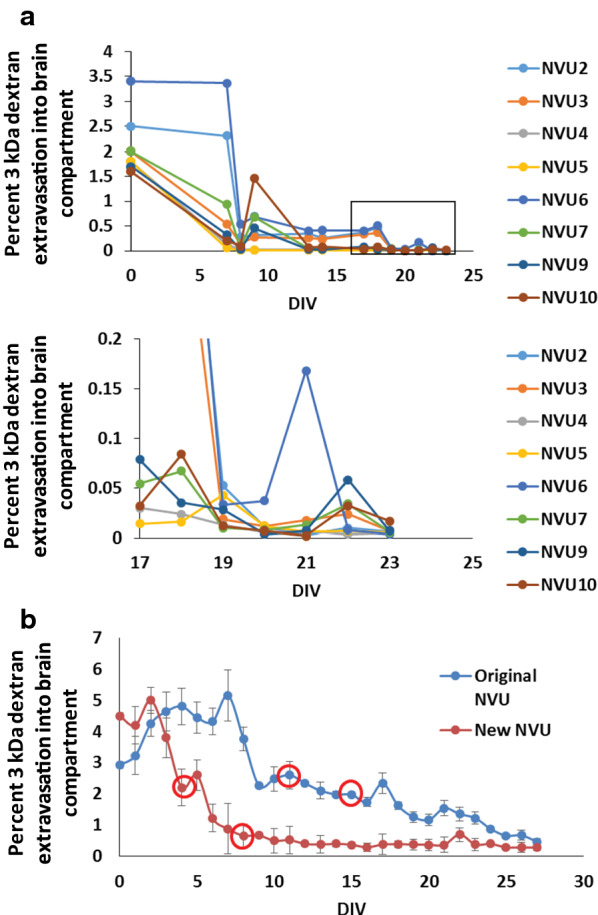
Timeline for barrier formation in NVUs seeded with primary human BMECs. **a** Time course for barrier formation in the new NVU design as measured by 3 kDa dextran extravasation. Each curve represents a single NVU device (N = 8 biological replicates). The top panel contains the full dataset, while the bottom panel is the magnified inset to highlight the permeability values on the y-axis at later time points. **b** Explicit comparison of barrier formation between the original and new NVUs. The first red circle indicates when primary human astrocytes were added to the brain chamber. The second red circle indicates when iPSC-derived cortical neurons were added to the brain chamber. Because neurons did not improve barrier properties, they were not utilized in further experiments. Data represent mean ± S.E.M. from N = 10 biological replicates

**Fig. 3:**
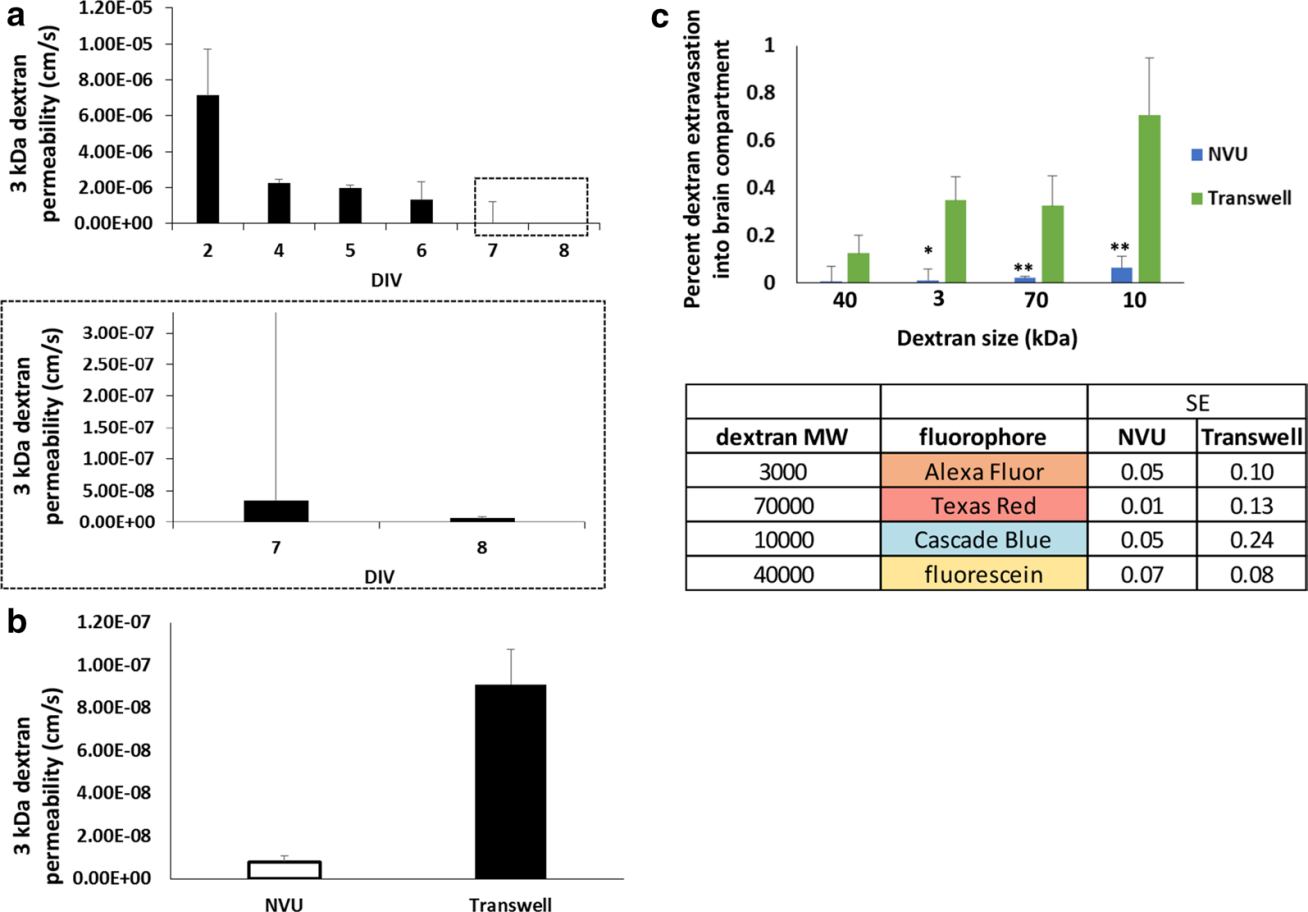
Permeability comparisons in primary human BMECs seeded in NVUs or Transwell setups. **a** Explicit permeability calculations for 3 kDa dextran extravasation through primary human BMECs in NVUs. The top panel contains the full dataset, while the bottom panel is the magnified inset to highlight the permeability values on the y-axis at later time points. Data represent mean ± S.E.M. from N = 10 biological replicates. **b** 3 kDa dextran permeability comparison between primary human BMECs seeded in NVUs versus Transwell filters. Measurements were acquired on day 8 after BMECs were seeded in each platform. Data represent mean ± S.E.M. from N = 10 biological replicates. Statistical significance was calculated using the student’s unpaired t-test (**, p < 0.01). **c** Comparison of size-dependent dextran permeability between primary human BMECs seeded in NVUs versus Transwell filters. Measurements were acquired on day 8 after BMECs were seeded in each platform. The corresponding table notes the size of each dextran and its corresponding fluorophore. The table also notes the standard error from each experimental system. Data in the bar chart represent mean ± S.E.M. from N = 10 biological replicates. Statistical significance was calculated using the student’s unpaired t-test (*p < 0.05; **p < 0.01). For all experiments, astrocyte co-culture was initiated on day 1

In the previous iteration of our NVU device, primary human BMECs required a relatively long time course for barrier development on the order of 12–14 days [23]. In an attempt to shorten this time, we made several changes to our setup and approach. First, we employed differential serum exposure (serum on the vascular side, no serum on the brain side), whereas we had previously used serum in both compartments [23]. Second, we switched from a polycarbonate membrane to a polyethylene terephthalate membrane of different pore size (which also improved live imaging capabilities; Fig. 1). Third, we seeded astrocytes earlier into the brain chamber on day 4 instead of day 11. Collectively, these changes further shortened the time to barrier formation to 8 days (Fig. 2a). A direct comparison to our old NVUs demonstrated increased barrier tightness at day 12 (Fig. 2b). The data collected in Fig. 2 initially used a 40 kDa dextran to assess permeability, and we also examined a smaller 3 kDa dextran and observed the same accelerated barrier formation (Fig. 3a). We further compared permeability between the NVUs and Transwells at day 8 and determined that the NVUs produced a tighter barrier with respect to 3 kDa dextran permeability (Fig. 3b). Thus, optimization of NVU device design with primary BMECs improved barrier formation within this simplified BBB model.

After these initial optimizations, we comprehensively compared barrier function in primary BMECs on Transwells and NVUs using fluorescent dextrans of varying molecular size (3 kDa up to 70 kDa). 3 kDa, 10 kDa, and 70 kDa dextran showed significantly lower permeability in the NVUs compared to Transwells with membranes of the same material and pore size, whereas permeability to the 40 kDa dextran was consistently lower in the NVUs but the difference relative to Transwells was not statistically significant (Fig. 3c). When comparing experimental variability between Transwells and NVUs, the variance between NVUs was roughly half that of Transwells when comparing standard error using the same number of samples (Fig. 3d). Thus, the NVU devices are highly consistent and can develop a tight passive barrier in this basic BBB model.

### Evaluation of P-glycoprotein function in the NVU model

**Fig. 4:**
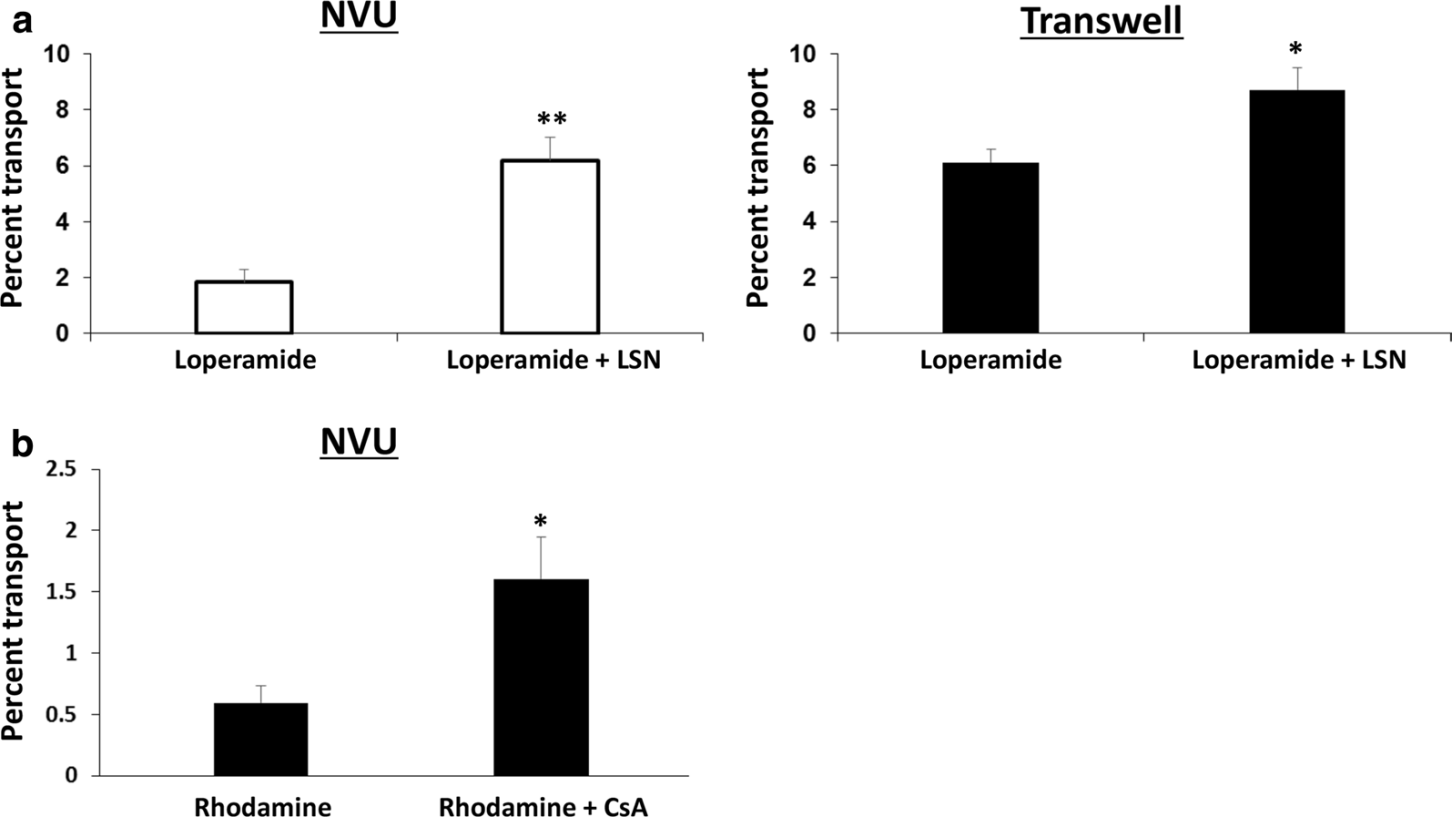
P-glycoprotein efflux activity in NVUs. Data in **a** were acquired with primary human BMECs cultured for 8 days prior to measurements. Data in **b** were acquired with iPSC-derived BMECs cultured for 2 days prior to measurements. For all experiments, astrocyte culture was initiated on day 5 for primary BMEC and day 1 for iPSC-derived BMECs. **a** Inhibition of P-glycoprotein in primary human BMECs via LSN335984 increases loperamide permeability regardless of culture format. Data represent mean ± S.E.M. from N = 5 biological replicates. Statistical significance was calculated using the student’s unpaired t-test (*p < 0.05; **p < 0.005). **b** Inhibition of P-glycoprotein in iPSC-derived BMECs via cyclosporin A (CsA) increases rhodamine permeability in NVUs. Data represent mean ± S.E.M. from N = 5 biological replicates. Statistical significance was calculated using the student’s unpaired t-test (*p < 0.05)

We next evaluated P-glycoprotein efflux activity in the optimized NVU model. We initially tested loperamide, which has poor CNS penetration because it is a substrate for P-glycoprotein. After pre-treatment with the P-glycoprotein inhibitor LSN335984 (provided by Eli Lilly and Company) [31], increased transport of loperamide was observed across a monolayer of primary BMECs; a similar outcome was observed across primary BMECs seeded on Transwells, but the difference was less pronounced (3.6-fold in the NVUs versus 1.4-fold in Transwells) (Fig. 4a). We also tested rhodamine 123 transport across iPSC-derived BMECs and observed the expected increase in permeation after pre-treatment with the P-glycoprotein inhibitor cyclosporin A (Fig. 4b); these results were consistent with many previous studies that have used iPSC-derived BMECs in Transwells [25]. Brightfield images demonstrated that BMEC monolayers were not disrupted by inhibitor treatment (Additional file 1: Figures S1 and S2), and immunocytochemistry confirmed VE-cadherin + endothelial cells with robust tight junction formation (Additional file 1: Figure S3). Thus, regardless of BMEC source, efflux activity is maintained in the NVU for this basic BBB model.

### Evaluation of opioid transport in the NVU model in response to P-glycoprotein inhibition and cortisol treatment

**Fig. 5:**
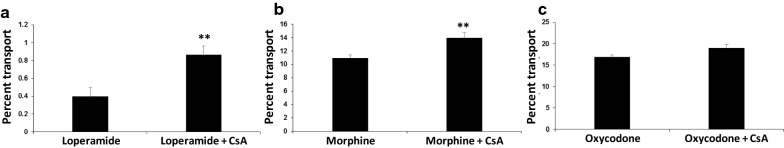
Drug transport across iPSC-derived BMECs cultured in NVUs. Data were acquired with iPSC-derived BMECs cultured for 2 days prior to measurements. For all experiments, astrocyte culture was initiated on day 1. Permeability was assessed for loperamide (**a**), morphine (**b**), and oxycodone (**c**) with and without P-glycoprotein inhibition by CsA. Data represent mean ± S.E.M. from N = 5 biological replicates. Statistical significance was calculated using the student’s unpaired t-test (*p < 0.05)

**Fig. 6:**
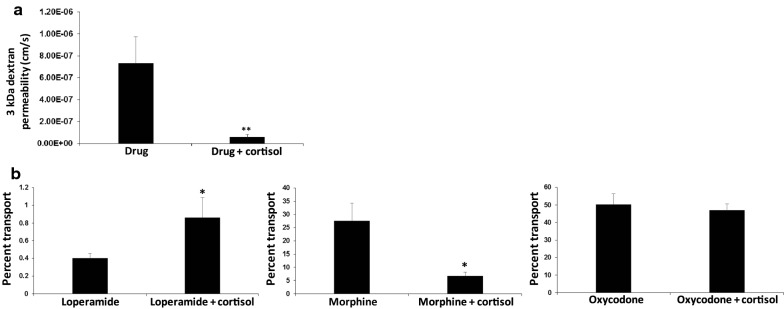
Influence of cortisol on drug transport across iPSC-derived BMECs cultured in NVUs. Data were acquired with iPSC-derived BMECs cultured for 2 days prior to measurements. For all experiments, astrocyte culture was initiated on day 1. **a** When measuring the permeability of any drug, 3 kDa dextran was included such that passive permeability could be simultaneously measured. These data in **a** represent pooled dextran permeability measurements for loperamide, morphine, and oxycodone with and without cortisol treatment (mean ± S.E.M. from N = 15 biological replicates). Statistical significance was calculated using the student’s unpaired t-test (**p < 0.005). **b** Permeability of loperamide, morphine, and oxycodone with and without cortisol treatment. Data represent mean ± S.E.M. from N = 5 biological replicates. Statistical significance was calculated using the student’s unpaired t-test (*p < 0.05)

Having validated P-glycoprotein activity, we proceeded to use iPSC-derived BMECs to examine the permeability in the NVU model of three different opioids with high, medium, and low P-glycoprotein sensitivity: loperamide, morphine, and oxycodone, respectively. As expected, permeability of loperamide and morphine was increased in the presence of cyclosporin A, whereas permeability of oxycodone was unchanged (Fig. 5). Next, we evaluated the effect of cortisol on opioid permeability. As expected from previous studies, cortisol treatment significantly reduced 3 kDa dextran permeability after 24 h of exposure (Fig. 6a). However, opioid transport profiles after 24 h of cortisol treatment were rather surprising. Transport of loperamide was significantly increased by cortisol treatment (Fig. 6b). In contrast, morphine transport was significantly decreased by cortisol treatment, whereas oxycodone transport was not influenced by cortisol treatment (Fig. 6b). These transport trends were consistent with experiments performed in Transwell filters, although the results were no longer statistically significant (Additional file 1: Figure S4). The contrasting transport profiles between loperamide and morphine suggest that the effects of cortisol are at least partially independent of P-glycoprotein activity, which was corroborated by qPCR experiments demonstrating minimal change to *ABCB1* expression in the presence of cortisol (Additional file 1: Figure S5). Thus, canonical opioid transport profiles were recapitulated in the NVU model and we uncovered a prospectively novel role for cortisol in the regulation of opioid transport across BMECs.

## Discussion

Blood–brain barrier models constructed with Transwells have been used extensively for transport experiments over the last three decades. New technologies have been developed that recapitulate physiologic properties such as fluid flow in an effort to better assess transport and improve the accuracy of BBB testing platforms. Here, we improved upon the original design of our NVU device, using primary human BMECs for rapid optimization. This optimization led to improved imaging capabilities and faster barrier formation. We demonstrated that NVU devices seeded with primary BMECs have significantly lower passive diffusion and decreased variance relative to Transwells with similar pore size membranes. We also demonstrated that efflux activity is maintained in BMECs seeded in NVU devices. After optimization, we moved on to more biomimetic iPSC-derived BMECs and demonstrated that these cells could properly model opioid transport in the NVU devices. Last, we revealed that cortisol has a previously unrecognized ability to modulate opioid transport. A direct comparison of opioid transport in the microfluidic NVU model versus Transwell filters demonstrated that both systems yielded the same transport trends, but the microfluidic model was able to pick up relevant statistical differences, thereby highlighting its utility for transport studies (which is further discussed below). It is of course worth noting that microfluidic NVU models require specialized expertise to construct and are likely to be more expensive than Transwell setups, but if their predictions are more sensitive or accurate, such trade-offs might be favorable for future studies.

As expected, opioid transport was properly modeled in the microfluidic NVU model, which is a simplified version of the BBB containing BMECs and astrocytes. Moreover, the relative permeability of opioids with varying affinity for P-glycoprotein was in line with their expected potency (e.g. loperamide having very little analgesic activity whereas morphine and oxycodone having high analgesic activity). However, we were surprised to find that cortisol yielded divergent effects on transport of two opioids with varying affinity for P-glycoprotein. Cortisol is referred to as hydrocortisone when supplied as a medication or for research purposes, and it has been frequently used to increase passive barrier function within in vitro BMEC models [32–34], but the influence of cortisol treatment on transport of small molecules has not been extensively studied. In vivo, cortisol concentration in the bloodstream is naturally regulated by diurnal rhythm but can also become elevated by stress and influenced by various diseases [35]. Cortisol itself is a P-glycoprotein substrate [36, 37], suggesting that elevated cortisol levels could directly occupy P-glycoprotein and increase BBB penetration of other P-glycoprotein substrates such as opioids. Cortisol also signals through the glucocorticoid nuclear receptor, which is highly expressed in nearly every cell type in the body including BMECs [38]. Thus, we suspect that cortisol may influence opioid transport through a combination of P-glycoprotein occupancy and modulation of transcriptional responses in BMECs, particularly given our observation of divergent trends for loperamide and morphine transport in the presence of cortisol without large changes to *ABCB1* expression. It is also possible that cortisol modulates astrocyte behavior and that our observations in drug transport are due to the collective effects of cortisol on both BMECs and astrocytes. These results are intriguing given the recent observations that P-glycoprotein activity and other mechanisms for BBB transport may be controlled by diurnal rhythm [39, 40], which may reflect not only a dependency on cortisol but other hormones.

Our results also highlight the utility of the microfluidic NVU model for studying transport mechanisms. We have previously used our NVU model to trace metabolic responses to inflammatory cytokines, which were conducted with primary BMECs [22]. Our updated model design, which was optimized again with primary BMECs, helped reduce experimental variance and produce a tighter barrier. These designs paved the way for using iPSC-derived BMECs, which are recognized as having higher-fidelity barrier properties compared to primary BMECs. Overall, our observed passive and active barrier functions within microfluidic NVUs seeded with iPSC-derived BMECs compare favorably to another recent microfluidic model that utilized hypoxia to strengthen barrier formation [41]. We also note a recent iteration of a microfluidic NVU model constructed with primary cells that was used to model methamphetamine transport and metabolism [42]. Our model could be used in a similar manner to not only study opioid transport but also activity and potency, particularly if iPSC-derived pericytes, astrocytes, and neurons were used to construct a patient-specific isogenic model as described for Transwell systems [43]; indeed, as pericytes and neurons may also regulate transporter expression in BMECs, their future inclusion would be necessary to make predictions within the microfluidic model as accurate as possible. Beyond our demonstration of reduced variability, microfluidic NVUs also offer the capability to cycle perfusate and expose the vascular chamber to rhythmic concentrations of desired soluble factors, although a more complex perfusion system beyond a simple syringe pump would be required. Thus, as described above, a full isogenic NVU model could be used to test how natural hormone fluctuations collectively regulate BBB transport and activity of opioids and other drugs of abuse. Relative to animal studies that typically rely on single time points for analysis, in vitro models offer the ability to sample many time points in the context of realistic variables, which should be of significant utility when attempting to understand how natural biology and patient-specific risk factors such as stress levels may influence drug activity.

## Conclusions

We conclude that microfluidic NVU models can be used to model transport of drugs of abuse, and that cortisol, despite its known ability to improve passive barrier function, has an unexpected influence on drug permeation. Our work benchmarks the use of microfluidic NVU models for more comprehensive studies of drug penetration into the CNS and suggests that future models should seek to incorporate biologically-relevant cues that may have an underappreciated impact on drug transport.

## Supplementary information

**Additional file 1: Figure S1.** Cell fidelity in the presence of LSN335984. The vascular chamber contains primary human BMECs and the brain chamber contains primary human astrocytes. No evidence of cell death was observed in either condition. Scale bars, 400 μm. **Figure S2.** Cell fidelity in the presence of cyclosporin A. The vascular chamber contains human iPSC-derived BMECs and the brain chamber contains primary human astrocytes. No evidence of cell death was observed in either condition. Scale bars, 400 μm. **Figure S3.** Immunofluorescent labeling of BMECs. Expression of claudin-5, ZO-1, and VE-cadherin in iPSC-derived BMECs (panels A–C) and primary BMECs (panels D–F). **Figure S4.** Influence of cortisol on drug transport across iPSC-derived BMECs cultured in Transwell filters. Permeability of loperamide, morphine, and oxycodone with and without cortisol treatment. Data represent mean ± S.E.M. from N = 3 biological replicates. Differences in transport were not statistically significance by student’s unpaired t-test (p > 0.05). **Figure S5.** Relative expression of *ABCB1* in response to cortisol treatment in iPSC-derived BMECs cultured in well plates. *ABCB1* expression is decreased by ~ 1.25-fold after 24 h of cortisol treatment. Data represent mean ± S.D. from biological triplicates. Statistical significance was calculated using the student’s unpaired t-test.

## Data Availability

Not applicable.

## Abbreviations

BBB: Blood–brain barrier
BMEC: Brain microvascular endothelial cell
NVU: Neurovascular unit
CNS: Central nervous system
iPSC: Induced pluripotent stem cell
ECM: Extracellular matrix
RA: Retinoic acid
TCC: Thermostatted column compartment
MRM: Multiple-reaction monitoring
CsA: Cyclosporin A
