# Supplementary material for: Advances in blood–brain barrier modeling in microphysiological systems highlight critical differences in opioid transport due to cortisol exposure
Source: Fluids Barriers CNS. 2020 Jun 3;17:38. doi: 10.1186/s12987-020-00200-9 (PMC7269003; doi:10.1186/s12987-020-00200-9)
Supplement: Supplementary file 1 — Additional file 1: Figure S1. Cell fidelity in the presence of LSN335984. The vascular chamber contains primary human BMECs and the brain chamber contains primary human astrocytes. No evidence of cell death was observed in either condition. Scale bars, 400 μm. Figure S2. Cell fidelity in the presence of cyclosporin A. The vascular chamber contains human iPSC-derived BMECs and the brain chamber contains primary human astrocytes. No evidence of cell death was observed in either condition. Scale bars, 400 μm. Figure S3. Immunofluorescent labeling of BMECs. Expression of claudin-5, ZO-1, and VE-cadherin in iPSC-derived BMECs (panels A–C) and primary BMECs (panels D–F). Figure S4. Influence of cortisol on drug transport across iPSC-derived BMECs cultured in Transwell filters. Permeability of loperamide, morphine, and oxycodone with and without cortisol treatment. Data represent mean ± S.E.M. from N = 3 biological replicates. Differences in transport were not statistically significance by student’s unpaired t-test (p > 0.05). Figure S5. Relative expression of ABCB1 in response to cortisol treatment in iPSC-derived BMECs cultured in well plates. ABCB1 expression is decreased by ~ 1.25-fold after 24 h of cortisol treatment. Data represent mean ± S.D. from biological triplicates. Statistical significance was calculated using the student’s unpaired t-test. [file 12987_2020_200_MOESM1_ESM.docx]

**Additional file**

Advances in blood-brain barrier modeling in microphysiological systems highlight critical differences in opioid transport due to cortisol exposure

Jacquelyn A. Brown, Shannon L. Faley, Yajuan Shi, Kathleen M. Hillgren, Geri A. Sawada, Thomas K. Baker, John P. Wikswo, and^,^ Ethan S. Lippmann


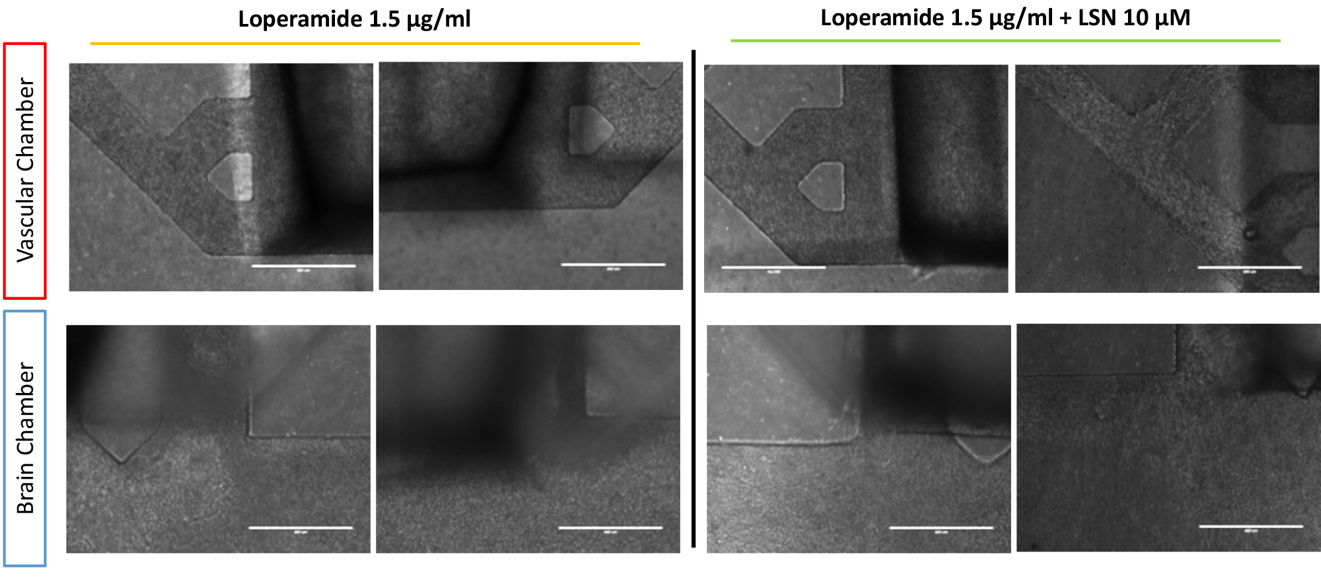


**Figure S1. Cell fidelity in the presence of LSN335984.** The vascular chamber contains primary human BMECs and the brain chamber contains primary human astrocytes. No evidence of cell death was observed in either condition. Scale bars, 400 μm.

**
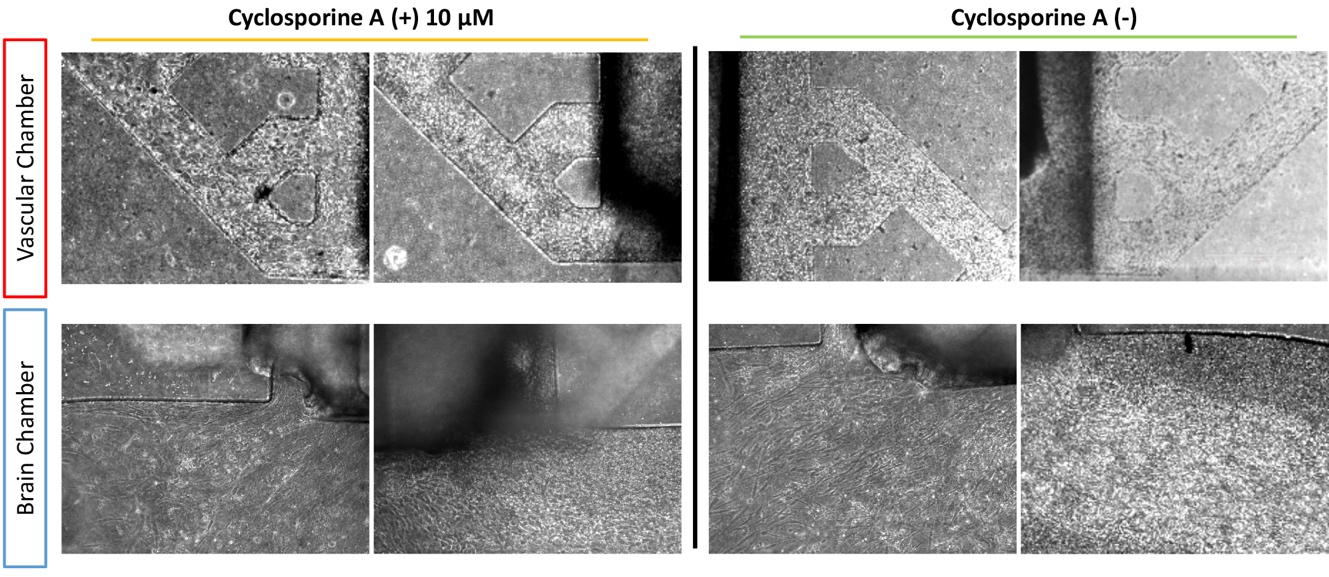
**

**Figure S2. Cell fidelity in the presence of cyclosporin A.** The vascular chamber contains human iPSC-derived BMECs and the brain chamber contains primary human astrocytes. No evidence of cell death was observed in either condition. Scale bars, 400 μm.

**
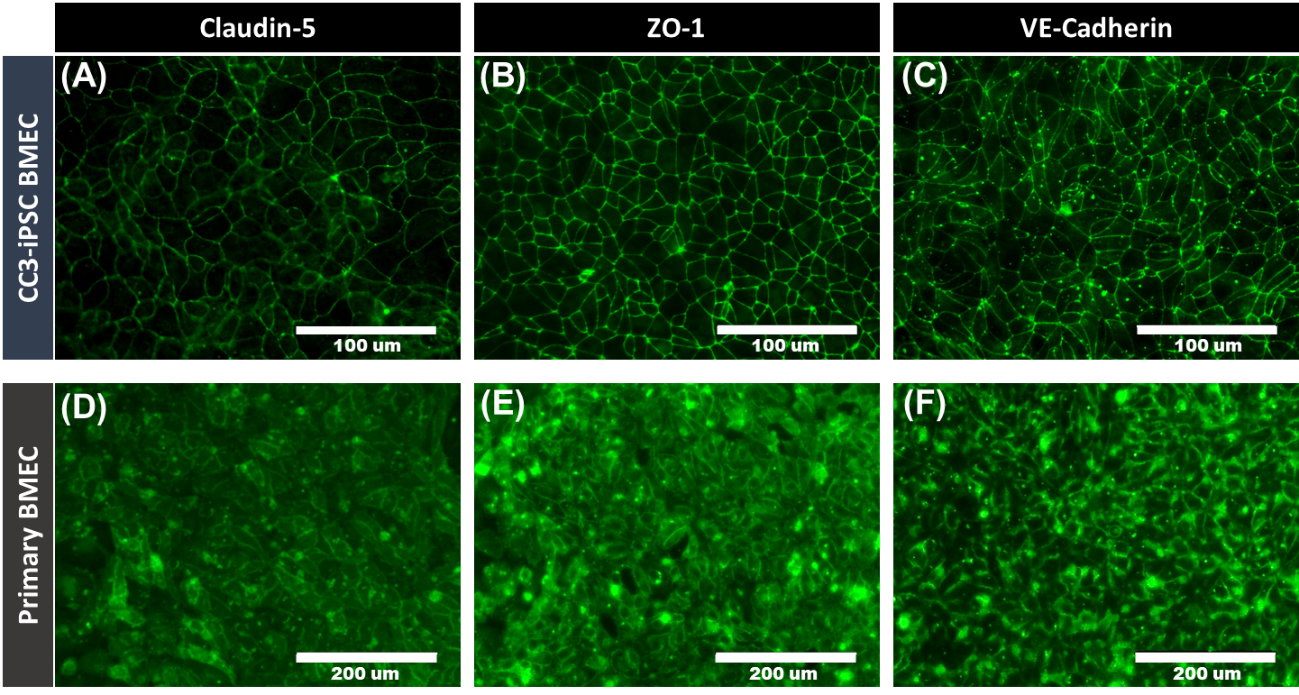
**

**Figure S3**. **Immunofluorescent labeling of BMECs.** Expression of claudin-5, ZO-1, and VE-cadherin in iPSC-derived BMECs (panels A-C) and primary BMECs (panels D-F).


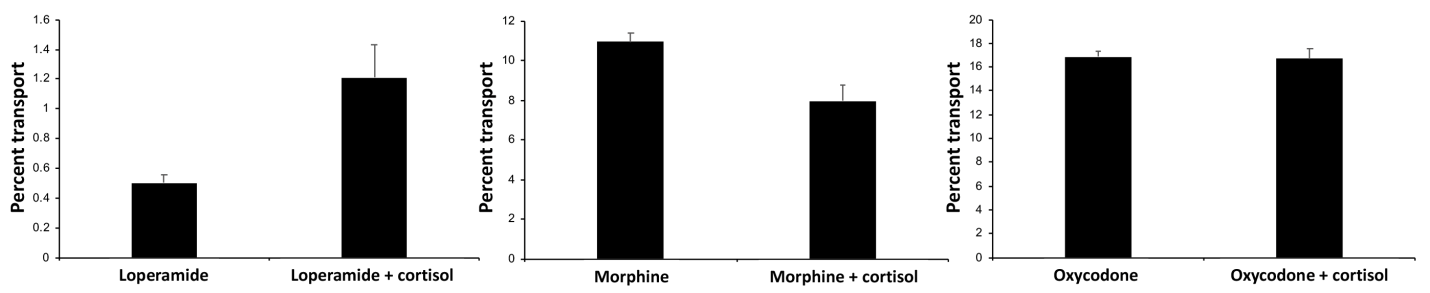


**Figure S4**. **Influence of cortisol on drug transport across iPSC-derived BMECs cultured in Transwell filters**. Permeability of loperamide, morphine, and oxycodone with and without cortisol treatment. Data represent mean ± S.E.M. from N=3 biological replicates. Differences in transport were not statistically significance by student’s unpaired t-test (p>0.05).


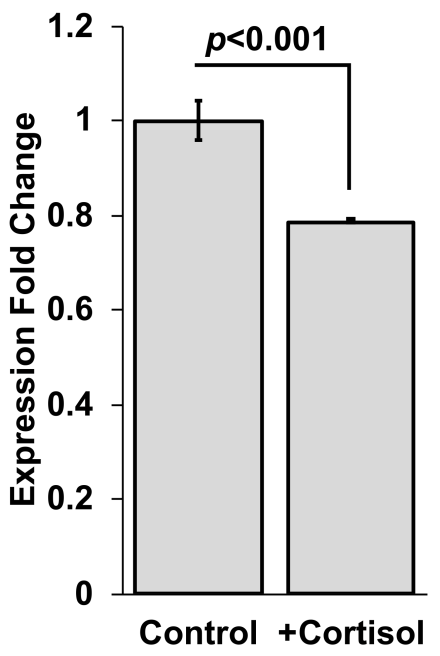


**Figure S5. Relative expression of *ABCB1* in response to cortisol treatment in iPSC-derived BMECs cultured in well plates.** *ABCB1* expression is decreased by ~1.25-fold after 24 hours of cortisol treatment. Data represent mean ± S.D. from biological triplicates. Statistical significance was calculated using the student’s unpaired t-test.
